# RT-qPCR Normalization Genes in the Red Alga *Chondrus crispus*

**DOI:** 10.1371/journal.pone.0086574

**Published:** 2014-02-03

**Authors:** Nathalie Kowalczyk, Sylvie Rousvoal, Cécile Hervé, Catherine Boyen, Jonas Collén

**Affiliations:** 1 Centre National de la Recherche Scientifique, UMR7139 Végétaux marins et biomolécules, Station Biologique de Roscoff, Roscoff, Brittany, France; 2 Université Pierre et Marie Curie - UPMC Paris 6, Station Biologique de Roscoff, Roscoff, Brittany, France; University of Melbourne, Australia

## Abstract

*Chondrus crispus* is a common red macroalga living on the rocky shores of the North Atlantic Ocean. It has a long research history, being a major source of carrageenan, a thickener widely used in the food industry, but also for physiological and ecological studies. To establish it as a model for red algae, its genome has been sequenced, allowing the development of molecular tools such as quantification of gene expression, including RNAseq and RT-qPCR. To determine appropriate genes for RT-qPCR normalization, the expression of 14 genes was monitored in 18 conditions using two sets of algal samples: samples from the sequenced strain, cultured and stressed in laboratory conditions and *C. crispus* collected on the shore and stressed *in situ*. The expression stability of the genes between the samples was evaluated by comparing the Ct range and using the programs geNorm and NormFinder. The candidate genes encoded translation related proteins (initiation factors IF4A-1 and IF4A-2, elongation factor EF1*α* and eRF3, an eukaryotic polypeptide chain release factor), cytoskeleton proteins (two *β*-tubulins, *α*-tubulin and actin), enzymes involved in the pentose phosphate pathway (glucose 6-phosphate deshydrogenase), protein recycling process (ubiquitin and ubiquitin-conjugating enzyme) and glycolysis (isocitrate dehydrogenase). The two sets of samples showed different expression patterns. Most of the genes were stable in the algae cultivated in the laboratory, whereas environmental samples showed a more important variation in gene expression. When analyzing the two sets separately, the ranking of the most stables genes were different from one method to another. When considering all samples, the two statistical methods were concordant, revealing translation initiation factor 4A-2 and eukaryotic polypeptide chain release factor 3 as pertinent normalization genes. This study highlights thus the importance of testing reference genes according to the experiments as well as the genetic and physiological background of the organism.

## Introduction

*Chondrus crispus* is a red macroalga, widely represented in the intertidal and subtidal zones of the rocky shores of the North Atlantic Ocean. Because of its ecological abundance and its economical importance, it has been one of the principal sources for carrageenan, a gellifying and thickening molecule used in the food industry, it also has a relatively important research history. Its habitat is a dynamic environment undergoing rapid changes of large amplitude in physical and chemical parameters due to the tidal cycles, combined with diurnal and weather variations. Studies about stress in *C. crispus* have been carried out, first with physiological approaches [1] [2] [3], then with molecular tools such as large scale quantitative transcriptomics [4] [5].

In previous reports [4] [6] [7], RT-qPCR has been used for targeted expression studies in *C. crispus*. This technique is sensitive to quantify gene expression and highly reliable [8] [9], but depends on the stability of the genes used as reference for data normalization. Different methods of identifying normalization genes have been developed so far, such as geNorm [10], NormFinder [11] and BestKeeper [12]. These algorithms have been used in many reports, in a wide range of organisms and tissues, first in animals (dolphin [13], fish [14], worm [15]), then in plants (rice [16], grapevine [17], banana [18]) and more recently in the brown algal model *Ectocarpus siliculosus* [19].

Until now, actin has been used as a normalization gene for *C. crispus*, based on the use of this gene as a common reference gene in other species. However, the stability of the expression of this gene has not been tested in *C. crispus*. To our knowledge, few other studies in red algae have used RT-qPCR for gene expression, however, when performed, usually without data normalization [20] [21]. In one case, a gene with an unknown function was used as a reference [22], and in one recent study, Wu *et al.* showed that *β*-tubulin was an appropriate normalization gene for expression studies between *Porphyra haitanensis* gametophyte and tetrasporophyte [23]. Even if normalization genes have been defined in many species, it appears that there is no universal gene common to every organism. Quantitative transcriptomics is an important part of the work on *C. crispus* and RT-qPCR aims to exploit accurately the high-throughput data. The genome of *C. crispus* has been sequenced and annotated [24], simplifying the determination and identification of the optimal normalization genes.

## Materials and Methods

### Culture conditions and treatments

The collections of algae were made on public property and according to French law no permission is needed for the collection of limited amounts of seaweed for non-commercial purposes. *Chondrus crispus* is a common seaweed, is not considered endangered and is not a protected species.

The red alga *Chondrus crispus* Stackhouse (Gigartinales, Rhodophyta) from two different origins was used for the experiments (see table 1), the samples A, F, G, H, I and M were gametophytes collected near the port of Bloscon in Roscoff (48.724, −3.970 Brittany, France). The algae labelled C1 to C12 were samples from the strain of *C. crispus* “Peggy's Cove”, a gametophyte collected in 1986 in Peggy's Cove, Canada and kept in 10 L plastic flasks in filtered and autoclaved NSW (natural sea water) in a culture room at 13°C and bubbled with compressed air. Light was provided by fluorescent tubes with a photon flux density of 100 µmol of photons·m^2^·s^−1^ for 12 hours per day.

**Table 1:** Culture conditions, treatments and duration.

Sample	Treatment	Duration
A	100% NSW - 100% sunlight	0 h
F	200% NSW - 35% sunlight	3 h
G	100% NSW - 35% sunlight	3 h
H	200% NSW - 100% sunlight	3 h
I	100% NSW - 100% sunlight	3 h
M	100% NSW - 100% sunlight	5 h
C1	NSW	3 h
C2	10 mM H_2_O_2_	3 h
C3	200 mg·L^−1^ harpin	3 h
C4	200 µM CdCl_2_	3 h
C5	200 µM CuSO_4_	3 h
C6	200 µM ZnCl_2_	3 h
C7	200 µM AlCl_3_	3 h
C8	500 µM dichlorvos	3 h
C9	500 µM glyphosate	3 h
C10	500 µM paraquat	3 h
C11	NSW+1% DMSO	3 h
C12	100 µM methyl jasmonate in DMSO (1%)	3 h

Culture conditions and duration. NSW: natural sea water, DMSO: Dimethyl sulfoxide.

For the chemical treatments the algae were transferred into Petri dishes containing 5 mL of NSW and the additives for 3 hours. The treatments were 10 mM H_2_O_2_, 200 mg·L^−1^ harpin, 200 µM CdCl_2_, 200 µM CuSO_4_, 200 µM ZnCl_2_, 200 µM AlCl_3_, 500 µM dichlorvos, 500 µM glyphosate, 500 µM paraquat, 1% DMSO and 100 µM methyl jasmonate with DMSO (1%).

The treatments for the field collected algae were full sunlight in NSW for 0, 3 and 5 h to follow the diurnal cycle. For hypersaline stress combined with light stress, algae were exposed to full sunlight in enriched NSW with 33 g·L^−1^ of NaCl added (200% NSW), sunlight filtered to 35% in NSW and sunlight filtered to 35% in NSW enriched with 33 g·L^−1^ of NaCl. All samples were immediately frozen in liquid nitrogen and stored at −80°C for 12 months.

Three biological replicates, each containing a pool of algal thalli, were obtained for each treatment and these were used for RNA extraction.

### Nucleic acid extractions

RNA and DNA were extracted using respectively Qiagen RNeasy plant kit and DNeasy plant kit, according to the manufacturer's protocols with the following two modifications: 100 mg of frozen tissue were ground in liquid nitrogen and were resuspended in the extraction buffer. After mixing vigorously for several minutes, a centrifugation step was added to eliminate cellular debris. After the RNA extraction, a treatment with RNAse-free DNAse I (Turbo DNAse, Ambion) was performed in order to eliminate residual genomic DNA.

### RNA quantification and cDNA synthesis

Nucleic acid concentrations were measured by absorbance at OD_260_ using a Thermo NanoDrop 2000 spectrophotometer. The purity of RNA samples was assessed by measuring the ratio OD_260_/OD_280_ and OD_230_/OD_260_ (see table S1 and table S2). RNA integrity was verified by capillary electrophoresis using the Agilent Bioanalyzer 2100 (Fig. S1), according to the manufacturer's instructions or by agarose gel electrophoresis (Fig. S2). The RNA of each sample was diluted and 800 ng was reverse-transcribed to cDNA using oligo(dT)_12–18_ and the Superscript II RT kit (InVitrogen) according to the manufacturer's protocol, and subsequently diluted with nuclease free water to 0.5 ng µL^−1^.

### Real-time PCR

For each gene, a pair of oligonucleotide sequences was designed close to the 3′ end of the coding sequence using Primer Express from Applied Biosystems (Table 2). Primers were designed to have a melting temperature around 60°C, a length between 18–26 nucleotides, a GC content between 40–60%, and avoiding secondary structures and self- and cross-annealing. The specificity of the oligonucleotides was tested *in silico*, using BLAST on the whole genome of *C. crispus*. The Q-PCR reactions were performed in a 96-well Chromo 4 thermocycler (Bio-Rad) with SYBRgreen PCR master kit. The protocol was: 14 min at 95°C, followed by 40 cycles of 15 s at 95°C and 60 s at 60°C. Each sample was technically triplicated. *C. crispus* genomic DNA was used as a quantification reference. A 1∶6 dilution series ranging from 29 to 37,500 copies of the *C. crispus* genome was prepared and tested for each gene in order to determine the amplification efficiency (equations of the standard curves in the Data S1). The specificity of the amplification was verified with a dissociation curve obtained by heating the samples from 65°C to 95°C. In addition to the DNAse I treatments, the absence of genomic DNA was confirmed by attempting to amplify an intron sequence using the cDNA as a template. The number of copies of contaminant gDNA (less than 100 copies) was subtracted from all other values, prior to any further analysis.

**Table 2:** Sequences of the primers used for quantitative PCR in *C. crispus*.

Gene code	Accession number	5′ (forward) - 3′ (reverse)	E (%)	R^2^	Tm product	PRL (bp)
IF4A-1	XM_005714063.1	CCCGACAAACCGTGAGAAC	99	0.997	82.57	82
		CAAGAAGTTAATGGCGACACC				
UBQ	XM_005711099.1	TCAGACTACAACATCCAGAAGG	100	0.9945	85.87	126
		CTTACGGCACACCATACGG				
eRF3	XM_005712129.1	GAGGAGTTCACCGTGTCTAAG	94	0.9975	81.46	117
		CGACATCTTCAACTGTACTAAGC				
Tub *β*-1	XM_005714305.1	TTGCCGCCACCTTTATCG	91	0.995	83.54	137
		GAACTCCATCTCATCCATACCC				
G6PdH	XM_005717558.1	AAACGGACCTGAAAGTAGTAATG	105	0.9645	83.32	114
		ATGCCCAAACACGAGAACC				
EF1*α*	XM_005712045.1	CTCCGCAGCAACCATTCG	103.5	0.9945	82.35	91
		AGCATGACCACTGTGTTACC				
IF4A-2	XM_005718687.1	CTCGCCTCTGCCACATTC	99.5	0.994	83.23	100
		GCCTTGTCGTCCTGGTAATC				
UbCE	XM_005713333.1	GCCACCAGTTGTCAAGTTTG	100	0.9975	79.88	80
		GAGGATGTCCAAGCAGATACC				
Tub *β*-2	XM_005715615.1	CGACCTTGTTTCCGAATATCAG	98	0.9855	80.17	84
		TCGTTCTCATACCCTTCTTCTTC				
IcdH	XM_005711636.1	AATCCGATTGCCTCTATATTTGC	101	0.995	80.3	66
		GGTCCCATCCAACTTTCCTC				
Actin	XM_005717205.1	ATTTGACTGCCTTTAACTCCATC	99	0.998	80.61	135
		GGGTCTCAATCTCCTTCTGC				
Tub-*α*	XM_005713236.1	TGCTCAATCGCCAACTCG	98	0.999	82.33	113
		ATACCCTCGCCCACATACC				
NUox	XM_005716764.1	AGTGCCATACACGATTCTGC	100	0.998	82.8	72
		AACAACAGCGAGTCCATCC				
Sulf	XM_005713481.1	TTTGAGAATCGCACTGGTAAAC	89	0.968	85.13	133
		ATACTCCTTATCCATCCACTCTG				
Intron	XM_005711099.1	GCTTGGGTTTCGCACATTAC	100	0.999	85.27	146
		GCATCAAGAAGAAGATTAAGCCT				

E: efficiency, R^2^: correlation coefficient, PRL: PCR product length, bp: base pair.

## Results and Discussion

### Treatments and choice of housekeeping genes

Two sets of conditions were tested in this study. In one set, algae collected on the shore were submitted to treatments *in situ* immediately after sampling in order to reproduce conditions close to natural stresses. As the diurnal rhythm has a strong influence on metabolism and physiology of red algae, a series of samples collected at three different times of the day was also analyzed. In tide pools, light, temperature and salinity are known to vary considerably, with subsequent changes in the gene expression. The environmental samples had a genetic background different from the sequenced strain from which the PCR primers were designed. The comparison of environmental samples with cultured samples represents the originality of this study.

The other set of experiments was carried out in laboratory controlled conditions, using samples of the sequenced strain of *C. crispus*. The agents used such as H_2_O_2_, a reactive oxygen species produced by many organisms, including algae, under conditions of abiotic and biotic stress, as well as methyl jasmonate, are known to induce a strong stress response at the transcriptomic level in *C. crispus* [4]. Metals like copper, cadmium, zinc and aluminium were also tested, being important pollutants in marine environments. Harpin, dichlorvos, paraquat (methyl viologen) and glyphosate are widely used pesticides able to induce a strong expression of stress response genes [6]. Paraquat and glyphosate are herbicides, the first generates reactive oxygen species by re-rooting electrons from the photosystem I to molecular oxygen and the other alters the structure of cell wall polysaccharides.

RNA was extracted from biological triplicates of algae treated as above, resulting in a total of 54 samples for 18 conditions. The abundance of the transcripts of 14 potential housekeeping genes was assayed. First, candidate genes considered as normalization genes for RT-qPCR in other marine eukaryotic and prokaryotic species were chosen [19] [25]. Then, among this list, 12 genes have been selected from a transcriptomics experiment done on environmental samples of *C. crispus* and correspond to the most stably expressed genes in this experiment (unpublished data). The candidate genes encoded translation related proteins (initiation factors IF4A-1 and IF4A-2, the elongation factor EF1*α* and eRF3, an eukaryotic polypeptide chain release factor), cytoskeleton proteins (two *β*-tubulins, *α*-tubulin and actin), an enzyme involved in the pentose phosphate pathway (glucose 6-phosphate deshydrogenase), proteins involved in the protein degradation and recycling process (ubiquitin and ubiquitin-conjugating enzyme) and also and enzyme involved in glycolysis (isocitrate dehydrogenase). Two additional genes were also selected for their high differential expression as negative controls: a NADH-ubiquinone oxidoreductase and a Galactose-2,6-sulfurylase (table 2).

### Quantification and data analysis

**Figure 1:**
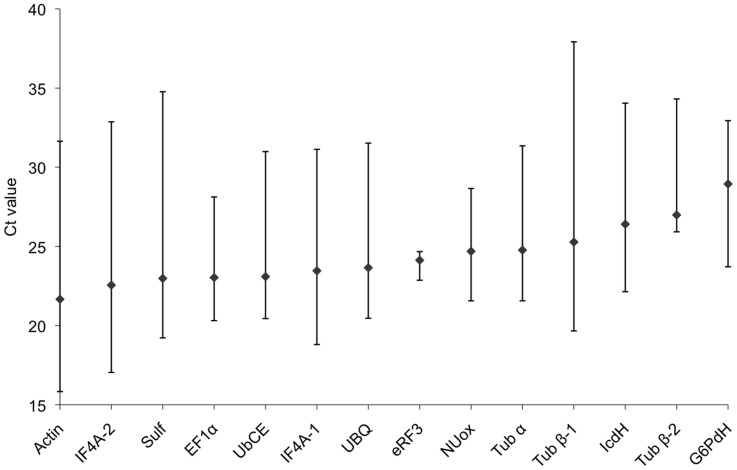
Ct values of the 14 housekeeping genes considering all tested samples. The range of expression of the 14 candidate genes are represented by the average Ct values (diamonds) and Ct ranges (bars).

The first analysis aimed to assess whether the transcript levels of the candidate genes were comparable between the different conditions. The cycle threshold (Ct [9]) value variations have been calculated for each gene and are shown in Fig. 1 (and Fig. S1). All genes had different levels of expression, corresponding to Ct-range from 21.66 to 28.94, and some of them were influenced by the treatments. *Actin* and *IF4A-2* showed higher average expression values than the other genes, while *G6PdH* had the lowest expression. Most of the genes had more than 7 cycles of variation between the samples and one gene, *eRF3*, was equally expressed in all samples and showed very little variation in the Ct values with only 1.81 cycles of variation.

When considering the two sets of samples separately, the variations in the Ct range were smaller, 7 genes from the “cultured” set showed less than 3 cycles of variations (see supplementary data Fig. S3A), *eRF3* was still stable but at the second position (1.81 cycles), the smallest Ct value variation was showed by *Tub β-2* (1.16 cycles). A similar shrinking was observed for the field samples (supplementary data Fig. S3B) were *eRF3* and *Tub β-2* had the same rank and showed even smaller variations in Ct values (1.5 and 0.56 cycles respectively).

**Figure 2:**
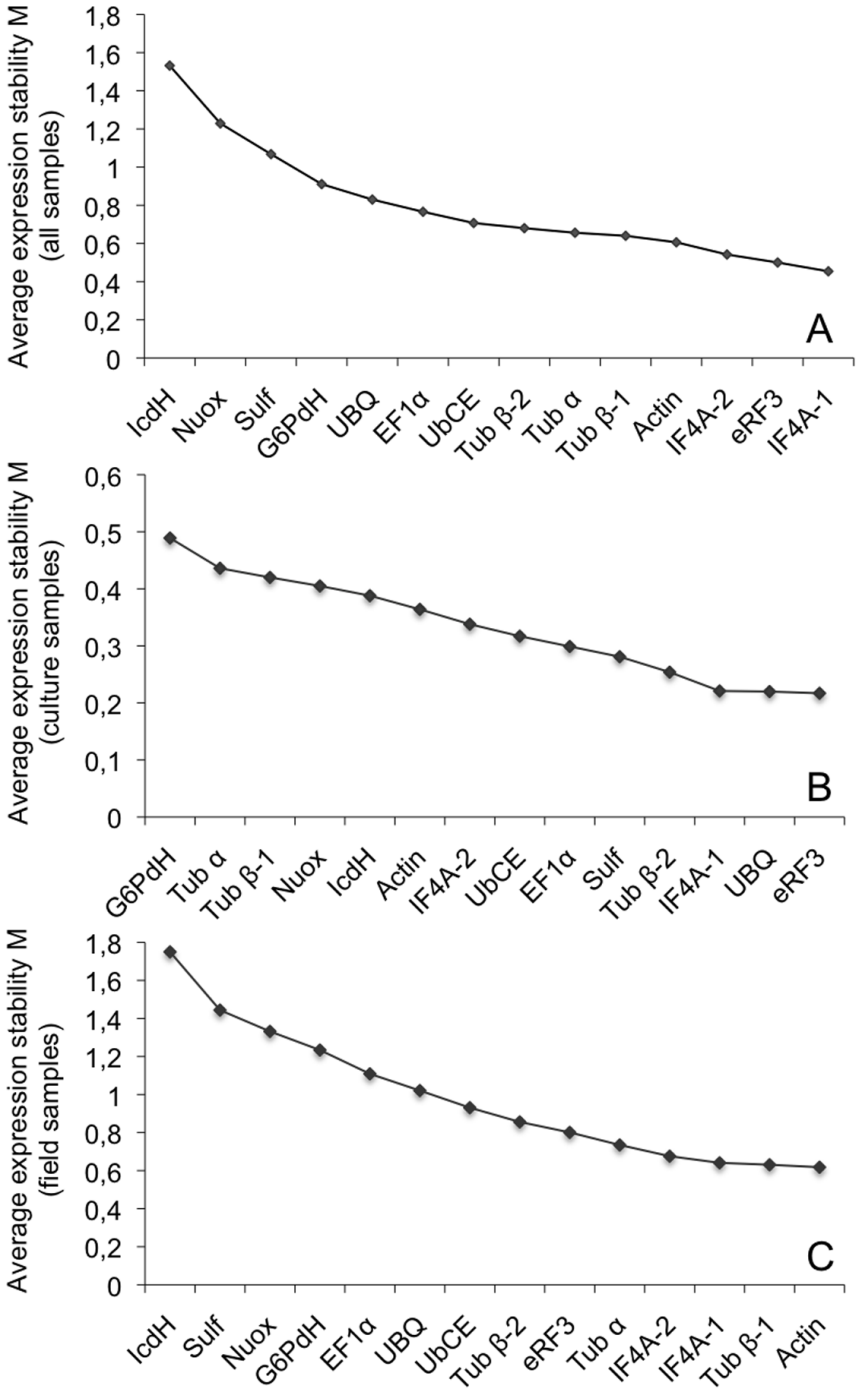
M value analysis of the expression stability of the 14 housekeeping genes. The 14 genes are ranked according to their M value, calculated by the geNorm software [10]. Low values of M inidicate that a gene is expressed very stably. A. all samples, B. culture samples, C. field samples.

The geNorm pairwise analysis was performed to test the robustness of the data. This analysis was first described by Vandesompele *et al.* [10] and is still widely used to evaluate normalization genes. The stability of the genes was tested between the different conditions and the results are shown in Fig. 2A. Genes were considered suitable when M was inferior to 0.5. Two genes fulfilled this criterium: *eRF3* and *IF4A-1*, the most stable being *IF4A-1*. The least stable genes were *IcdH*, *Nuox* and *Sulf*. The M values were also calculated for the two sets of samples separately. For the culture samples (Fig. 2B) the most stable genes were *eRF3*, *UBQ* and *IF4A-1*. For the field samples (Fig. 2C) the genes were different: *actin*, *Tub β-1* and *IF4A-1*.

**Figure 3:**
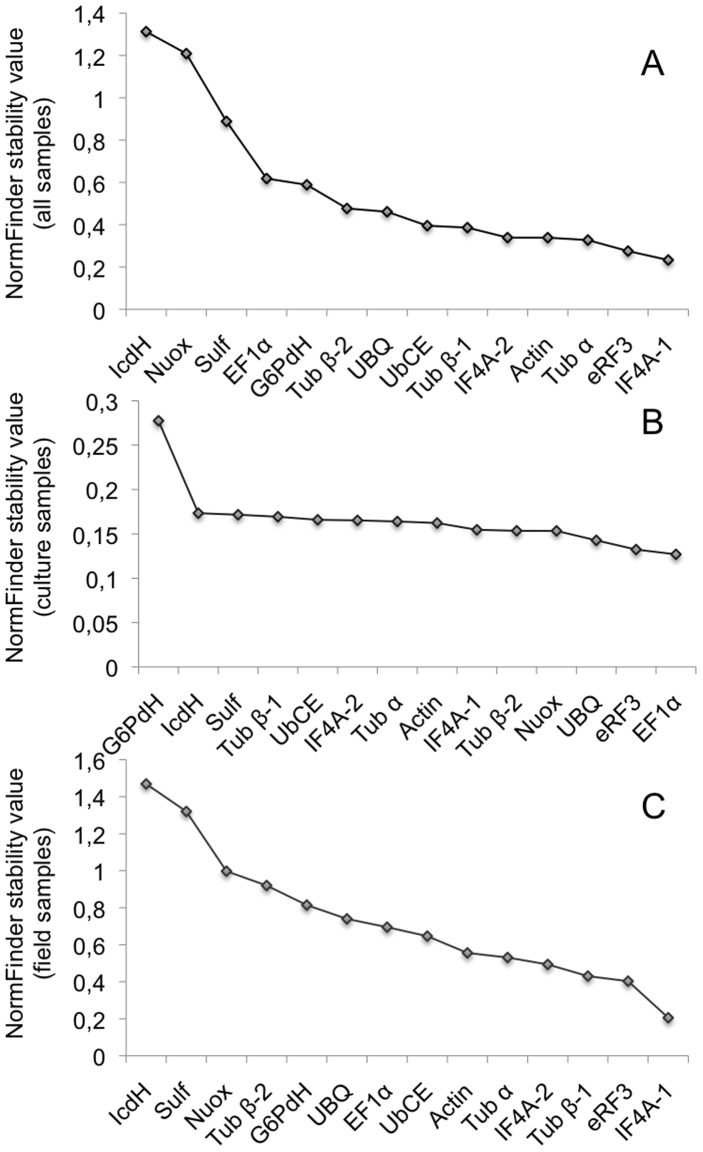
NormFinder analysis of the expression stability of the 14 housekeeping genes. The stability of expression of the 14 candidate genes was calculated using the NormFinder method [11]. A. all samples, B. culture samples, C. field samples.

Another approach, using NormFinder, was also used to test the candidate genes (Fig. 3A). The ranking of the genes with intermediate M values was different, compared to the geNorm analysis. However, there was a good correlation for the two most stable genes (*eRF3* and *IF4A-1*) and the three most fluctuating. To calculate a normalization factor, NormFinder showed that the best combination of two genes was *eRF3* and *actin*, with a stability value of 0.195. Interestingly, when the analysis was done with culture samples the ranking was different (Fig. 3B), *eRF3* and *EF1α* being the most stable, but with the environmental samples (Fig. 3C) the same genes as the full analysis were considered as most stable.

**Figure 4:**
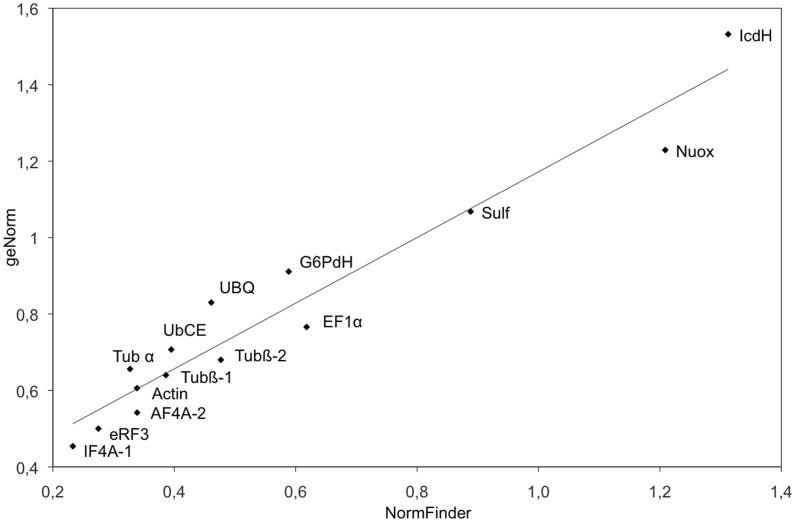
NormFinder analysis vs. geNorm analysis of the 14 housekeeping genes. The stability of expression of the 14 candidate genes was compared between the NormFinder method (abscissa axis) and the geNorm method (ordinate axis). (R^2^ = 0.94).

Plotting the geNorm and NormFinder analyses one against the other (Fig. 4) supports the global ranking of the 14 genes, for the whole set of samples.

## Conclusion

In this study, two methods were used to identify the best normalization genes : geNorm and NormFinder and the results were concordant. The experiment was made with two sets of samples, six samples harvested on the field and thus with a varied genetic and physiological background, and twelve samples corresponding to the laboratory-cultured sequenced strain of *C. crispus* thus having the same genetic and physiological background. The field samples came from an environment with dynamic conditions, due to tidal cycles and weather changes. The sequenced strain is cultured in very stable conditions. These two environments lead to different physiological responses to the stressing conditions to which the algae have been submitted. Field samples have a more plastic metabolism than the cultured algae, that were acclimated to stable conditions. When considering the complete data set, the large variations in Ct values are clearly due to the differences between the two sets of samples, which are more homogenous when considered separately than all together. The cultivated samples had less variation in Ct values than the samples from the field (see Data S2).

From the result of the geNorm analysis, the samples from Peggy's Cove exhibited a stable expression for all the candidate genes. The trend was similar with the NormFinder analysis, but since the differences between values was small, the ranking of the genes was slightly different. Thus if the experiment was carried out with only laboratory samples, as it is frequently done, all the tested genes could have been used as normalization genes, even those which were supposed to have differential expression. However, when considering the field samples, the stability values are higher and the ranking of the genes is different.

For expression studies concerning only environmental samples, even if the results were not fully concordant, a trend emerged, *Tub β-1* and *IF4A-1* were well ranked in the different methods used. When tallying all methods, *IF4A-1* and *eRF3* are the best candidate gene for normalization, as they have a good rank whatever the method used for calculations, especially when analyzing samples from various origins and having thus different behaviors.

## Supporting Information

Figure S1 **RNA Bioanalyzer gel for environmental samples.** Quality of RNA in environmental samples.
(TIFF)

Figure S2 **RNA gel for culture samples.** Quality of RNA in culture samples. Ladder range : 0.2–10 kb.
(PDF)

Figure S3 **Ct-range of the 14 housekeeping genes.** A. culture samples, B. field samples.
(TIFF)

Table S1 **RNA quantifications for environmental samples.** Concentration of RNA in environmental samples.
(PDF)

Table S2 **RNA quantifications for culture samples.** Concentration of RNA in culture samples.
(PDF)

Data S1 **Equations of the standard curves for both PCR runs.**
(TXT)

Data S2 **Ct values of all the samples.**
(XLS)
