# Supplementary figures and images for: RT-qPCR Normalization Genes in the Red Alga Chondrus crispus
Source: PLoS One. 2014 Feb 3;9(2):e86574. doi: 10.1371/journal.pone.0086574 (PMC3912222; doi:10.1371/journal.pone.0086574)

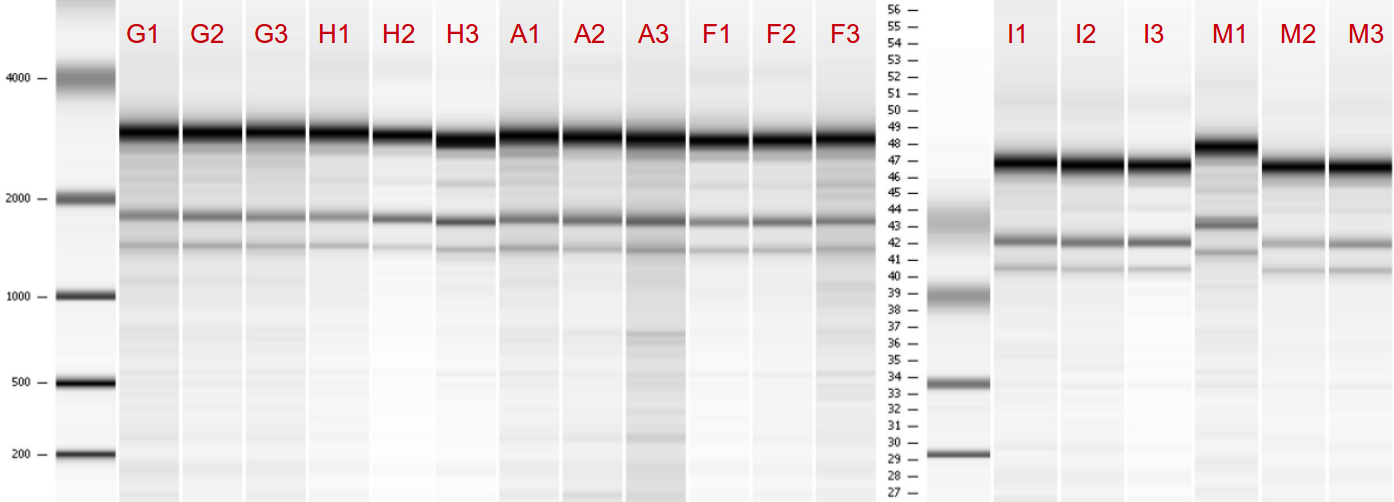

Supplement: Figure S1 — RNA Bioanalyzer gel for environmental samples. Quality of RNA in environmental samples. (TIFF) [file pone.0086574.s001.tif]

C1a C2a C3a C4a C5a C6a C7a C8a C9a C10a C11a C12a

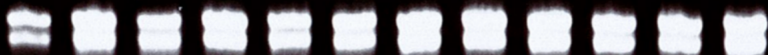

C1b C2b C3b C4b C5b C6b C7b C8b C9b C10b C11b C12b

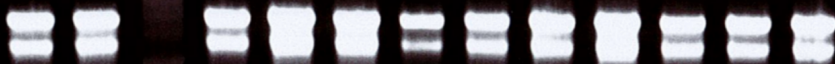

C1c C2c C3c C4c C5c C6c C7c C8c C9c C10c C11c C12c

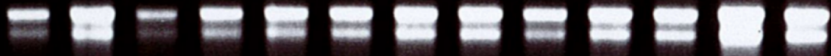

Supplement: Figure S2 — RNA gel for culture samples. Quality of RNA in culture samples. Ladder range : 0.2–10 kb. (PDF) [file pone.0086574.s002.pdf]

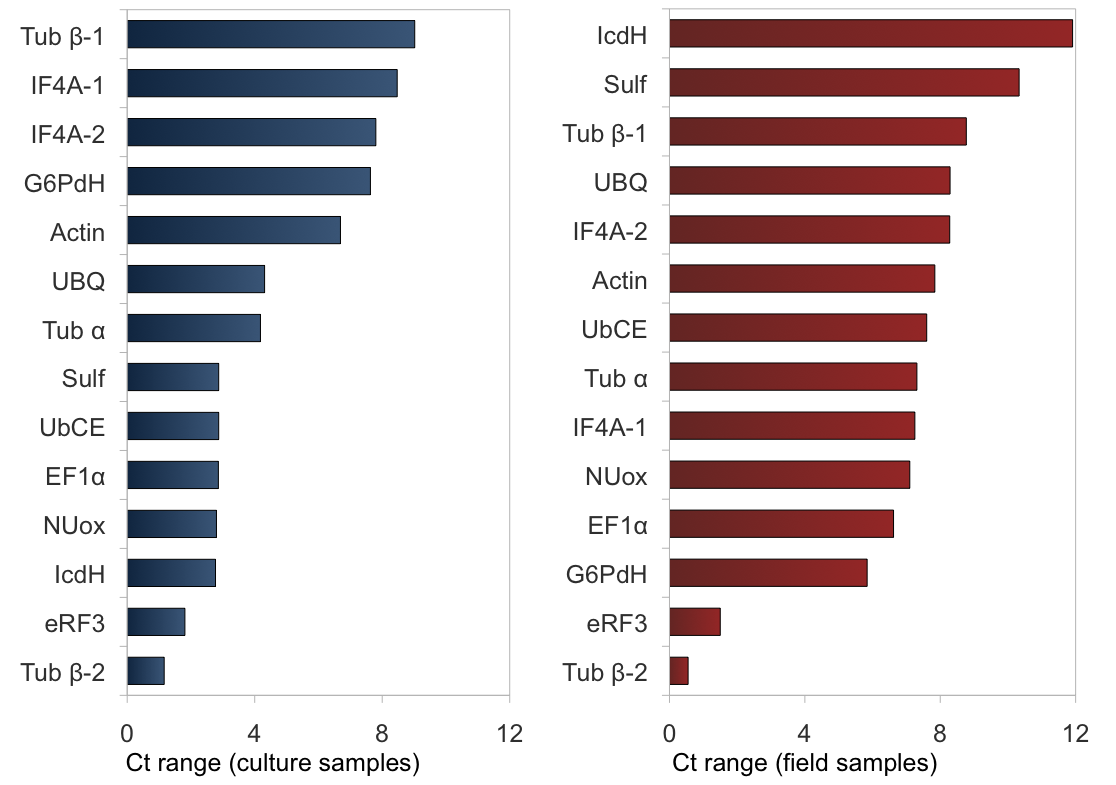

Supplement: Figure S3 — Ct-range of the 14 housekeeping genes. A. culture samples, B. field samples. (TIFF) [file pone.0086574.s003.tif]
