# Supplementary material for: RT-qPCR Normalization Genes in the Red Alga Chondrus crispus
Source: PLoS One. 2014 Feb 3;9(2):e86574. doi: 10.1371/journal.pone.0086574 (PMC3912222; doi:10.1371/journal.pone.0086574)
Supplement: Table S1 — RNA quantifications for environmental samples. Concentration of RNA in environmental samples. (PDF) [file pone.0086574.s004.pdf]

Sample	[RNA] $\mu g.\mu l^{-1}$	260/280	260/230
1A	0.4	1.9	1.72
2A	0.2	2.0	1.89
3A	0.2	2.0	1.78
1F	0.3	2.0	2.01
2F	0.2	2.0	1.95
3F	0.3	1.9	1.97
1G	0.3	2.0	2.00
2G	0.2	2.0	2.00
3G	0.3	2.0	1.99
1H	0.2	2.0	1.27
2H	0.3	2.0	1.97
3H	0.3	2.0	1.84
1I	0.2	2.0	1.85
2I	0.2	2.0	1.93
3I	0.2	2.0	1.86
1M	0.3	2.0	2.00
2M	0.1	2.0	1.75
3M	0.2	2.0	1.90
