# Supplementary material for: RT-qPCR Normalization Genes in the Red Alga Chondrus crispus
Source: PLoS One. 2014 Feb 3;9(2):e86574. doi: 10.1371/journal.pone.0086574 (PMC3912222; doi:10.1371/journal.pone.0086574)
Supplement: Table S2 — RNA quantifications for culture samples. Concentration of RNA in culture samples. (PDF) [file pone.0086574.s005.pdf]

Sample	[RNA] μg.μl^{-1}	260/280
C1a	1.5	2.0
C2a	2.2	2.0
C3a	2.0	2.0
C4a	2.2	2.0
C5a	1.6	1.9
C6a	2.2	2.0
C7a	2.4	2.0
C8a	2.5	2.0
C9a	2.5	2.0
C10a	2.0	2.0
C11a	2.1	2.0
C12a	2.3	2.0
C1b	2.4	2.2
C2b	2.4	2.2
C3b	2.3	2.2
C4b	2.9	2.1
C5b	2.3	2.2
C6b	1.1	2.2
C7b	1.7	2.1
C8b	2.2	2.2
C9b	2.7	2.2
C10b	2.0	2.2
C11b	1.9	2.2
C12b	2.2	2.1
C1c	0.6	2.1
C2c	1.1	2.1
C3c	0.4	2.1
C4c	0.7	2.1
C5c	0.9	2.2
C6c	0.8	2.1
C7c	1.0	2.1
C8c	1.1	2.1
C9c	0.9	2.2
C10c	1.0	2.1
C11c	0.8	2.1
C12c	1.6	2.1
